# Reply to: Does the generalized paroxysmal fast activity‐underrepresented cluster represent Lennox–Gastaut syndrome or other severe developmental and epileptic encephalopathies?

**DOI:** 10.1002/epi.70506

**Published:** 2026-09-18

**Authors:** Emanuele Cerulli Irelli, Marco Perulli, Maria S. Borioni, Adolfo Mazzeo, Carlo Di Bonaventura, Domenica I. Battaglia

**Affiliations:** ^1^ Department of Human Neurosciences Sapienza University Rome Italy; ^2^ Pediatric Neurology and Psychiatric Unit Fondazione Policlinico Universitario Agostino Gemelli IRCCS Rome Italy; ^3^ IRCCS Neuromed Pozzilli Italy

To the Editors,

We thank Dr. Fusco for her thoughtful comments on our study, “Refining Diagnostic Boundaries and Electroclinical Profiles of Lennox–Gastaut Syndrome Through Unsupervised Clustering.”^1^ Her letter provides an opportunity to clarify the scope, aims, and interpretation of our work and to address the specific issues raised.

Our objective was not to question the mandatory role of the electroencephalographic (EEG) criteria included in the current International League Against Epilepsy (ILAE) definition,^2^ nor to suggest that every developmental and epileptic encephalopathy (DEE) with tonic seizures should be classified as Lennox–Gastaut syndrome (LGS). Rather, we deliberately studied a clinically defined population characterized by childhood onset DEE, intellectual disability, tonic seizures, and at least one additional seizure type, and investigated how the data‐driven phenotypic structure within this population related to the current electroclinical definition of LGS.

From this perspective, we agree with Dr. Fusco that Cluster 1 should not be interpreted as establishing a novel LGS subtype and that our data do not support diagnostic equivalence between patients fulfilling and not fulfilling the complete ILAE criteria. This point was explicitly acknowledged in our article, where the clustering solution was described as a descriptive representation of multidimensional phenotypic structure rather than as an independent validation of discrete electroclinical subtypes.

The clinically relevant observation emerging from the unsupervised clustering was somewhat different and, in our view, was consistent with the discriminative value of current criteria within a clinically overlapping population, rather than providing evidence against them; patients who did not fulfill complete electroclinical criteria were not randomly distributed across the clusters, but were preferentially represented within a severe early onset profile characterized by profound developmental and motor impairment, previous epileptic spasms, EEG background slowing, structural etiologies, and comparatively infrequent generalized paroxysmal fast activity (GPFA; i.e., Cluster 1). Whether these patients represent incomplete expression of the LGS electroclinical phenotype, phenotypic convergence of other severe DEEs, or a partly distinct group cannot be determined from our retrospective data and requires longitudinal investigation. Additionally, our article explicitly cautioned that differences between clusters may partly reflect overall encephalopathic severity rather than discrete LGS subtypes, and our published discussion similarly emphasized that the GPFA‐underrepresented cluster should be regarded as hypothesis‐generating rather than as a distinct nosological entity.

For the same reason, we do not believe that repeating the unsupervised clustering after restricting the analysis to patients already fulfilling complete electroclinical criteria would constitute a direct sensitivity analysis of our original question. Such an analysis would address a different question, because it would remove precisely the electroclinical heterogeneity that our study was designed to characterize. Our objective was not to determine whether complete electroclinical LGS itself can be further subdivided, but to investigate how data‐driven phenotypic structure relates to current diagnostic boundaries within a clinically defined population.

Dr. Fusco also raises the related issue of therapeutic implications and clinical trial design. We agree that potential therapeutic benefit in patients with other severe DEEs does not necessarily require their inclusion within the LGS category.^3^ At the same time, criteria used to assign a formal syndromic diagnosis and those historically used to define populations in therapeutic trials are not necessarily identical. Several pivotal randomized trials of treatments now established for LGS—including rufinamide, clobazam, cannabidiol, and fenfluramine—selected patients predominantly according to the clinical seizure phenotype, particularly tonic/drop seizures, together with broader EEG features such as slow spike‐and‐wave activity, without requiring demonstration of GPFA.^4^, ^5^, ^6^, ^7^, ^8^ Thus, a substantial part of the therapeutic evidence base for LGS has been generated in populations not selected according to the current complete electroclinical definition. This issue was also raised in our original article, where we noted that contemporary LGS trials have not consistently required GPFA for enrollment.

This observation should not be interpreted as evidence that GPFA lacks diagnostic relevance, nor does it imply that patients with other DEEs without this EEG pattern should be reclassified as having LGS. Rather, it highlights the distinction between criteria used for formal syndromic classification and the populations in whom therapeutic efficacy has historically been investigated. In this setting, characterizing phenotypic heterogeneity among patients with clinically overlapping presentations may therefore remain relevant to future trial stratification, eligibility criteria, and analyses of treatment response.

We also agree with Dr. Fusco that no empirically validated temporal threshold establishes the lower biological boundary of GPFA specifically in LGS. Brenner and Atkinson used a ≥1‐s operational definition,^9^ and subsequent studies in genetic generalized epilepsies have conventionally distinguished generalized polyspike trains (GPT) lasting <1 s from GPFA lasting ≥1 s.^10^, ^11^, ^12^ However, an operational distinction should not be equated with evidence of a physiological discontinuity at exactly 1 s.

This issue is illustrated by the study of Dalic et al. in the ESTEL LGS cohort,^13^ in which GPFA was defined as generalized 10–30‐Hz activity lasting >250 ms and event duration was analyzed quantitatively. The median duration of individual GPFA events was 1.2 s, with an interquartile range of .9–1.6 s, meaning that the population of events contributing to this clinically informative quantitative biomarker extended across the conventional 1‐s boundary. Dalic et al. did not directly compare <1‐s and ≥1‐s events or establish a biological continuum; nevertheless, their approach illustrates that duration may be more appropriately considered as a quantitative dimension rather than evidence that biological significance begins at exactly 1 s. More recently, Lévi‐Strauss et al. similarly defined GPFA using a >250‐ms threshold and examined autonomic responses associated with individual events. Although clinically expressed GPFA was longer than subclinical events, GPFA duration poorly correlated with the magnitude of the associated autonomic response (*r* = .068), further indicating that the physiological impact of these discharges is not simply determined by discharge duration.^14^ Importantly, however, this cohort was diagnostically heterogeneous, including patients with LGS, focal epilepsy with GPFA, and neurodevelopmental syndromes with GPFA, and the study did not examine whether GPFA characteristics differed according to the underlying diagnosis.

Additional observations from generalized epilepsy cohorts also question a sharp GPT–GPFA separation. Conrad et al. described substantial co‐occurrence and morphological similarity between shorter GPT and longer GPFA and considered the 1‐s distinction somewhat arbitrary.^11^ Similarly, in our previous multicenter study, the majority of patients exhibited both patterns, without identifiable electrophysiological differences other than discharge duration.^12^ These findings cannot be directly extrapolated to LGS, but they support caution in interpreting the 1‐s cutoff as evidence that generalized fast discharges on either side of this boundary represent categorically distinct neurophysiological phenomena.

We therefore re‐examined discharge duration in our cohort. In the overall population, among the 73 of 105 patients classified as having GPFA, 56 (76.7%) had at least one discharge lasting ≥1 s, whereas 17 (23.3%) showed only discharges lasting .5–<1 s. Within the original clustering solution, generalized fast discharges lasting ≥1 s were observed in 36.6% (15/41) of patients in Cluster 1, 54.8% (23/42) in Cluster 2, and 92.9% (13/14) in Cluster 3. Thus, even when applying the ≥1‐s threshold, longer generalized fast discharges remained least prevalent in Cluster 1, consistent with the GPFA‐underrepresented profile identified in the original analysis.

Moreover, when patients with only .5–<1‐s discharges were compared with those showing ≥1‐s discharges, we did not identify clear differences in seizure types, epilepsy etiology, or age at onset, although patients with shorter discharges showed a pattern suggestive of greater encephalopathic burden, as reflected by higher proportions of severe intellectual disability, severe motor impairment, and EEG background slowing, together with a lower proportion of normal magnetic resonance imaging findings (Table 1). These observations do not establish equivalence between shorter and longer generalized fast discharges but further emphasize the need for studies specifically designed to determine the validity and biological meaning of the 1‐s threshold.

**TABLE 1:** Comparison of clinical characteristics between patients with GPFA lasting <1 s and ≥1 s.

Characteristic	GPFA <1 s, *n* = 17	GPFA ≥1 s, *n* = 56	*p*
Age at last follow‐up, years, median (IQR)	22 (12–34.5)	27.5 (16.25–41)	.43
Female sex, *n* (%)	11 (64.7%)	35 (62.5%)	1
GMFCS 4–5 motor impairment, *n* (%)	12 (70.6%)	25 (44.6%)	.09
Severe intellectual disability, *n* (%)	16 (94.1%)	43 (76.8%)	.16
Normal MRI, *n* (%)	2 (13.3%)	22 (40%)	.06
Monogenic etiology, *n* (%)	2 (11.8%)	12 (21.4%)	.49
Infantile seizure onset, *n* (%)	15 (88.2%)	41 (73.2%)	.32
Polymorphic seizures at last visit, *n* (%)	13 (76.5%)	41 (73.2%)	1.00
Spasms, *n* (%)	11 (64.7%)	29 (51.8%)	.41
Atonic seizures, *n* (%)	9 (52.9%)	25 (44.6%)	.58
Atypical absences, *n* (%)	11 (64.7%)	34 (60.7%)	1
Focal seizures, *n* (%)	11 (64.7%)	32 (57.1%)	.77
Tonic–clonic seizures, *n* (%)	11 (64.7%)	45 (80.4%)	.20
Myoclonic seizures, *n* (%)	5 (29.4%)	23 (41.1%)	.57
Slow EEG background, *n* (%)	16 (94.1%)	37 (66.1%)	.02
SSWD, *n* (%)	16 (94.1%)	53 (94.6%)	1.00
Longest seizure‐free interval, *n* (%)			.63
None	9 (52.9%)	37 (66.1%)	
Weeks	5 (29.4%)	11 (19.6%)	
Months	2 (11.8%)	7 (12.5%)	
ASM tried during follow‐up, mean (SD)	8.94 (4.29)	10.2 (3.6)	.54

Abbreviations: ASM, antiseizure medication; EEG, electroencephalographic; GMFCS, Gross Motor Function Classification System; GPFA, generalized paroxysmal fast activity; IQR, interquartile range; MRI, magnetic resonance imaging; SSWD, slow spike–wave discharge.

Lastly, Dr. Fusco proposes characterizing GPFA according to duration, frequency, burden, morphology, topography, and sleep stage dependence. We agree that multidimensional quantitative characterization of GPFA represents an important research direction. However, such an analysis requires operational standards that are currently unavailable. Although canonical GPFA is descriptively characterized by generalized or bilateral fast activity, often with frontocentral predominance and spike/polyspike morphology,^15^ quantitative operationalization of these features remains limited. To our knowledge, no validated quantitative criteria specify the minimum amplitude relative to background required to define an event, the degree of bilateral or frontocentral predominance necessary to establish its topography, or objective morphological criteria separating rhythmic fast activity from related polyspike patterns. Introducing amplitude, topography, and morphology as additional variables would therefore require the post hoc creation of new thresholds whose biological validity would be no better established than that of the duration threshold under discussion.

Moreover, GPFA was only one component of a multidimensional clustering analysis rather than the primary focus of a quantitative EEG study. Incorporating several additional, nonvalidated electrophysiological dimensions would result in increasingly sparse categories and a greater multiple‐testing burden. We therefore believe that attempting to derive and validate such criteria retrospectively within the present cohort could imply a degree of electrophysiological precision not supported by the available statistical and methodological framework. Prospective studies specifically designed to quantitatively characterize generalized fast activity, preferably using continuous measures and data‐driven approaches rather than multiple arbitrary dichotomies, are better suited to address this important question.

In summary, we fully agree that complete electroclinical criteria remain important for the formal diagnosis of LGS. Our study addressed a complementary question: how phenotypic heterogeneity is structured among patients with a clinically overlapping LGS phenotype and how this structure relates to current diagnostic criteria. Rather than challenging those criteria, our findings show that patients less likely to fulfill them were preferentially represented within a severe early onset phenotype whose nosological and biological significance remains to be established prospectively. Conversely, among patients more likely to fulfill complete electroclinical criteria, two phenotypic profiles emerged, differing primarily in developmental severity, seizure phenotype, epilepsy trajectory, and age at onset. We believe that defining and prospectively studying these patterns may help clarify both the biological heterogeneity of LGS‐related phenotypes and their potential relevance to therapeutic stratification.

## CONFLICT OF INTEREST STATEMENT

E.C.I. has received speaker's fees from Angelini Pharma and travel support from UCB pharma, Jazz Pharmaceuticals, and Angelini Pharma outside the submitted work. M.P. has received honoraria from Jazz Pharmaceuticals, outside the submitted work. M.S.B. has no conflict of interest to disclose. A.M. has received speaker's fees from Angelini Pharma outside the submitted work. C.D.B. has received speaker's or consultancy fees from UCB Pharma, Eisai, GW Pharmaceuticals, Jazz Pharmaceuticals, Angelini Pharma, Lusofarmaco, and Ecupharma outside the submitted work. D.I.B. has received honoraria from UCB, Jazz Pharmaceuticals, and Biocodex for participation on advisory boards and/or in conferences, outside the submitted work. We confirm that we have read the Journal's position on issues involved in ethical publication and affirm that this report is consistent with those guidelines.

## DISCLAIMER

The views expressed in this article are solely those of the authors and do not necessarily reflect the official position of *Epilepsia* or the International League Against Epilepsy.

## Data Availability

The data that support the findings of this study are available on request from the corresponding author. The data are not publicly available due to privacy or ethical restrictions.
